# Role of RANKL-induced osteoclast formation and MMP-dependent matrix degradation in bone destruction by breast cancer metastasis

**DOI:** 10.1038/sj.bjc.6600858

**Published:** 2003-04-15

**Authors:** T Ohshiba, C Miyaura, M Inada, A Ito

**Affiliations:** 1 Department of Biochemistry, School of Pharmacy, Tokyo University of Pharmacy and Life Science, 1432 Horinouchi, Hachioji, Tokyo 192-0392, Japan

**Keywords:** bone metastasis, bone resorption, osteoclast, matrix metalloproteinases, breast cancer

## Abstract

Bone metastasis of breast cancer induces severe osteolysis with increased bone resorption. Osteoclast differentiation regulated by the receptor activator of NF-*κ*B ligand (RANKL) in osteoblasts and matrix degradation induced by matrix metalloproteinases (MMPs) are thought to be involved in the process of bone resorption. When nude mice were inoculated with human breast cancer cells, MDA-MB-231(MDA-231), numerous osteoclasts resorbed bone and the degradation of the bone matrix markedly progressed in the femur and tibia with metastasis of the MDA-231 tumour. The expression of RANKL, MMP-13 and membrane-type 1-MMP mRNA was markedly elevated in bone with metastasis. When MDA-231 cells were cocultured with mouse calvaria, MDA-231 markedly induced bone resorption measured by calcium release from the calvaria, and the expression of RANKL, MMP-2 and MMP-13 was elevated in the calvaria after the coculture. The separation of MDA-231 from the calvaria using filter insert showed decreased bone resorption, suggesting that cell-to-cell interaction is essential for cancer-induced bone resorption. Adding MDA-231 cells to bone marrow cultures markedly induced osteoclast formation, and the expression of RANKL in osteoblasts was enhanced by contact with the cell surface of MDA-231 cells. These results indicate that RANKL-induced osteoclast formation and MMP-dependent matrix degradation are associated with osteolysis because of bone metastasis of breast cancer.

Bone is the most common site of metastasis in breast cancer. Tumour cells released from the primary focus infiltrate into the blood vessels and reach bone to form skeletal metastasis. Bone metastasis is accompanied by severe bone destruction, but the molecular mechanisms of the tumour-induced bone resorption are not fully understood. Previous studies have shown that bisphosphonate, an inhibitor of osteoclastic bone resorption, is a beneficial agent for the treatment of bone metastasis in patients with breast cancer (Hortobagyi *et al*, 1996). In an experimental metastasis model using human breast cancer cells, bisphosphonates such as ibandronate suppress bone metastasis by the promotion of apoptosis of osteoclasts (Hiraga *et al*, 2001). Using the same model, a neutralising antibody to parathyroid hormone-related protein (PTHrP) suppresses bone metastasis (Guise *et al*, 1996). PTHrP is a secreted peptide produced by tumours and stimulates osteoclastic bone resorption (Powell *et al*, 1991). Although PTHrP is one of the candidates for regulators in tumour-induced osteolysis, other factors should also be involved in the mechanism of bone resorption at the site of metastasis.

Recently, the mechanisms of bone resorption have been actively examined, and the discovery of the receptor activator of NF-*κ*B ligand (RANKL) has allowed elucidation of the mechanism of osteoclast differentiation in bone (Anderson *et al*, 1997; Wong *et al*, 1997; Lacey *et al*, 1998; Yasuda *et al*, 1998). Osteoblasts and bone marrow stromal cells express RANKL as a membrane-associated factor in response to bone-resorbing factors such as parathyroid hormone (PTH), interleukin-1 (IL-1) and prostaglandin E2 (PGE2). Osteoclast precursors possess RANK, a receptor for RANKL, and RANK–RANKL recognition induces the differentiation of the precursors into osteoclasts. In osteolysis associated with cancer metastasis, osteoclasts have to play an important role in the process of bone resorption. Recent studies have shown that OPG inhibits cancer-induced osteoclastogenesis and prevents tumour growth in bone (Morony *et al*, 2001; Zhang *et al*, 2001), suggesting that tumour cells use the RANKL : RANK axis to induce osteolysis.

Matrix metalloproteinase (MMP) is a family of proteolytic enzymes involved in the degradation of the extracellular matrix of various tissues including bone. More than 20 different mammalian MMPs have been identified, and divided into four subgroups; collagenase (MMP-1, MMP-8, MMP-13 and MMP-18), gelatinase (MMP-2 and MMP-9), stromelysin (MMP-3 and MMP-10) and membrane-type metalloproteinase (MMPs-14–17) (Mauviel, 1993; Nagase and Woessner, 1999; Vu and Werb, 2000). It is known that osteoblasts produce various MMPs such as MMP-1, MMP-2, MMP-13 (collagenase 3) and MMP-14 (MT1-MMP), and that osteoclasts selectively produce MMP-9 (MacDougall and Matrisian, 1995; Kusano *et al*, 1998; Westermarck and Kahari, 1999). We have reported that the induction of MMPs (such as MMP-2, -3 and -13) in osteoblasts is essential for bone resorption (Kusano *et al*, 1998). On the other hand, the roles of MMPs in infiltration and metastasis of tumour cells have been widely investigated (MacDougall and Matrisian, 1995; Nakahara *et al*, 1997; Westermarck and Kahari, 1999). MT1-MMP expressed in tumour cells activates proMMP-2 produced by stromal cells in the primary focus and at the metastatic site (Sato *et al*, 1994). Therefore, it is possible that MMPs are involved in the process of bone metastasis. However, the expression of MMPs in bone tissue with tumour metastasis and the roles of these enzymes produced by host cells in tumour-induced osteolysis have not been investigated as yet.

In this study, to clarify the role of RANKL-induced osteoclast formation and MMP-dependent matrix degradation in osteolysis because of bone metastasis, we examined the expression of RANKL and MMPs in bone with metastasis using the intracardiac injection of breast cancer cells. Expression of RANKL, MMP-2, MMP-13 and MT1-MMP was markedly elevated in bone with metastasis of breast cancer MDA-MB-231 cells *in vivo*. We established a new *in vitro* system for cancer-induced bone resorption using tumour cells cocultured with mouse calvaria, and found that cell-to-cell interaction between tumour cells and osteoblasts induced the expression of RANKL in osteoblasts, resulting in osteoclast formation. We suggest that RANKL-induced osteoclast formation and MMP-dependent matrix degradation are essential for osteolysis because of bone metastasis.

## MATERIALS AND METHODS

### Animals, cells and reagents

Female BALB/c nu/nu (nude) mice (5 weeks old), and *ddy* mice, day 2, day 5 and 6 weeks of age were obtained from Japan SLC Inc. (Shizuoka, Japan). A human breast cancer cell line, MDA-MB-231 (MDA-231), was obtained from American Type Culture Collection (Rockville, MD, USA). MDA-231 cells were cultured in *α*-modified MEM (*α*MEM), supplemented with 10% fetal calf serum (FCS) at 37°C under 5% CO_2_ in air. Recombinant human IL-1*α* (IL-1) and recombinant mouse OPG were purchased from Genzyme/TECHNE. BB94, a general inhibitor of MMPs, was kindly provided by British Biotech Pharm., Ltd (Oxford, UK).

### Intracardiac injections of MDA-231 in nude mice

MDA-231 cells (1 × 10^5^) were suspended in 0.1 ml of PBS and injected into the left heart ventricle of 5-week-old female nude mice with a 27-gauge needle under anaesthesia with pentobarbital. Animals were kept in our specific pathogen-free animal facilities for 6 weeks. All procedures were performed in accordance with institutional guideline for animal research at the Tokyo University of Pharmacy and Life Science.

### Histological and radiographic analyses of the femur and tibia

Radiographs of the femurs and tibiae were taken by soft X-ray (model CMB-2; SOFTEX, Tokyo, Japan). The femurs and tibiae were fixed with 70% ethanol and embedded in glycol methacrylate, and undecalcified 3-*μ*m sections were prepared and stained for tartrate-resistant acid phosphatase (TRAP) or haematoxylin–eosin (HE), as reported previously (Miyaura *et al*, 1997).

### Northern blot analysis

Total RNA was extracted from the femur, tibia, cultured calvaria and cultured mouse osteoblastic cells, using the acid guanidium–phenol–chloroform method (Chen *et al*, 1997). For Northern blotting, 20 *μ*g of total RNA was resolved using electrophoresis on a 1% agarose–formaldehyde gel and transferred onto a nylon membrane (Hybond-N, Amersham Pharmacia Biotech, Tokyo, Japan), which was then hybridised with a ^32^P-labelled cDNA probe, as reported previously (Chen *et al*, 1997). A 946-bp fragment of mouse RANKL cDNA and a 485-bp fragment of mouse MMP-13 were prepared using RT–PCR cloning and used as the probes (Kusano *et al*, 1998; Yasuda *et al*, 1998). Mouse MT1-MMP cDNA was kindly provided by Dr M Seiki, and a 656-bp fragment was used as the probe (Sato *et al*, 1994). The signals were densitometrically quantified using an NIH-image analyzer.

### RT–PCR analysis

cDNA was synthesised from 5 *μ*g of total RNA by reverse transcriptase (Superscript II Preamplification System, Life Technologies, Grand Island, NY, USA) and amplified using PCR. The primers used in PCR for the mouse RANKL gene were 5′-GAC TCG ACT CTG GAG AGT-3′ (sense primer) and 5′-GAG AAC TTG GGA TTT TGA TGC-3′ (antisense primer). The reaction conditions for PCR were 32 cycles, denaturation at 94°C for 45 s, annealing at 58°C for 45 s and extension at 72°C for 2 min. The primers used in PCR for the mouse cathepsin K gene were 5′-CTT GTG GAC TGT GTG ACT-3′ (sense primer) and 5′-AAC ACT GCA TGG TTC ACA-3′ (antisense primer). The reaction conditions for PCR were 25 cycles, denaturation at 94°C for 45 s, annealing at 55°C for 45 s and extension at 72°C for 2 min. The primers used in PCR for the mouse TRAP gene were 5′-TGA CAA GAG GTT CCA GGA-3′ (sense primer) and 5′-AGC CAG GAC AGC TGA GTG-3′ (antisense primer). The reaction conditions for PCR were 25 cycles, denaturation at 94°C for 45 s, annealing at 55°C for 45 s and extension at 72°C for 2 min. The primers used in PCR for the mouse glyceraldehyde-3-phosphate dehydrogenase (GAPDH) gene were 5′-TGA AGG TCG GTG TGA ACG GAT TTG GC-3′ (sense primer) and 5′-CAT GTA GGC CAT GAG GTC CAC CAC-3′ (antisense primer). The reaction conditions for PCR were 30 cycles, denaturation at 94°C for 45 s, annealing at 60°C for 45 s and extension at 72°C for 2 min. The PCR product was run on a 1.5% agarose gel and stained with ethidium bromide.

### Coculture of MDA-231 cells and mouse calvaria

*Ddy* mice (5 days old) were killed and their calvariae were aseptically harvested and dissected free of suture tissues. The calvariae were divided into halves and cultured for 24 h in 0.5 ml *α*MEM containing 1 mg ml^−1^ BSA. After preculture for 24 h, each half calvaria was cocultured with MDA-231 cells (1 × 10^5^) embedded in 21 *μ*l of type I collagen gel (0.8 mg ml^−1^) for the positioning of coculture in 24-well plates. After 5 days of the coculture, the calvaria with MDA-231 cells was fixed with 10% formalin and used for histological analysis of TRAP and HE staining. To determine the bone-resorbing activity in the coculture, the concentration of calcium in the conditioned medium was measured on day 5 with a calcium kit (calcium C-test Wako; Wako Pure Chemical. Osaka, Japan). The conditioned medium was also used for Western blot analysis and gelatin zymography for the detection of MMP-2, MMP-9 and MMP-13. After the coculture, MDA-231 cells in collagen gel were removed from the calvaria, and the remaining calvariae were used for the extraction of total RNA for Northern blot analysis. In some experiments, the calvaria was separated from MDA-231 cells embedded in collagen gel using a cell culture insert with an 8.0 *μ*m pore (FALCON, Becton Dickinson, Franklin Lakes, NJ, USA).

### Gelatin zymography

Gelatinase activity in the conditioned medium of the coculture of the calvaria and MDA-231 cells embedded in collagen gel was analysed by gelatin zymography (Kusano *et al*, 1998). Aliquots (10 *μ*l) were mixed with 5 *μ*l of nonreducing SDS–PAGE sample buffer, then subjected to SDS–PAGE using 10% polyacrylamide gels containing 0.6 mg ml^−1^ of gelatin. After electrophoresis, the gels were incubated for 1 h in washing buffer consisting of 50 mM Tris-HCl containing 5 mM CaCl_2_, 1 *μ*M ZnCl_2_ and 2.5% Triton X-100 to remove SDS, and then in the same buffer without Triton X-100 at 37°C for 1 h. The gels were stained with 0.1% (w v^−1^) Coomassie Brilliant Blue in 50% (v v^−1^) methanol, 10% (v v^−1^) acetic acid, and destained in a solution of 30% (v v^−1^) methanol and 1% (v v^−1^) formic acid. Enzyme activity was detected as a clear zone in a darkly stained background.

### Western blot analysis

An aliquot of the conditioned medium of the coculture of calvaria and MDA-231 cells embedded in collagen gel was subjected to SDS–PAGE using 10% polyacrylamide gels and the separated proteins were transferred to a polyvinylidene difluoride membrane (PVDF, Millipore, Tokyo, Japan). The membrane was first incubated for 18 h at 4°C with 5% skim milk in phosphate-buffered saline containing 0.1% Tween-20 to block nonspecific binding, and then incubated for 2 h with polyclonal rabbit anti-mouse MMP-13 antibody, kindly donated by Dr G Murphy. After incubation with horseradish peroxidase-conjugated donkey anti-rabbit IgG for 1 h, immunoreactive bands were detected with an ECL system (Amersham Pharmacia Biotech, Tokyo, Japan).

### Cultures of primary mouse osteoblastic cells and MDA-231 cells

Primary osteoblastic cells were isolated from 2-day-old mouse calvariae after five routine sequential digestions with 0.1% collagenase (Wako, Tokyo, Japan) and 0.2% dispase (Godo Shusei, Tokyo, Japan), as described previously (Chen *et al*, 1997). Osteoblastic cells collected from fractions 3 to 5 were combined and cultured in *α*MEM supplemented with 10% FCS at 37°C under 5% CO_2_ in air. In some experiments, the MDA-231 cells were fixed with 3% paraformaldehyde and washed three times with culture medium. On the layer of fixed-MDA-231 cells, osteoblasts and/or bone marrow cells were cultured.

### Osteoclast formation in coculture of mouse bone marrow cells and osteoblasts

Bone marrow cells (2 × 10^6^ cells) were isolated from 6-week-old *ddy* mice, and cocultured with the primary osteoblasts (1 × 10^4^ cells) in 0.5 ml of *α*MEM containing 10% FCS in 24-well plates. Primary osteoblastic cells were collected from newborn mouse calvariae as described above. To examine the effects of MDA-231 cells on osteoclast formation, MDA-231 cells (1 × 10^4^ cells) were added to the coculture system. The cultures were fed every 3 days by replacing 0.3 ml of the old medium with fresh medium. Reagents such as OPG were added at the beginning of the culture and each time the medium was changed. After being cultured for 8 days, the cells adhering to the well surface were stained for TRAP. The TRAP-positive multinucleated cells containing three or more nuclei per cell were counted as osteoclasts.

### Statistical analysis

The data are expressed as the means±s.e.m. The significance of differences was analysed using the Student's *t*-test.

## RESULTS

### Osteolysis due to bone metastasis of breast cancer cells

**Figure 1:**
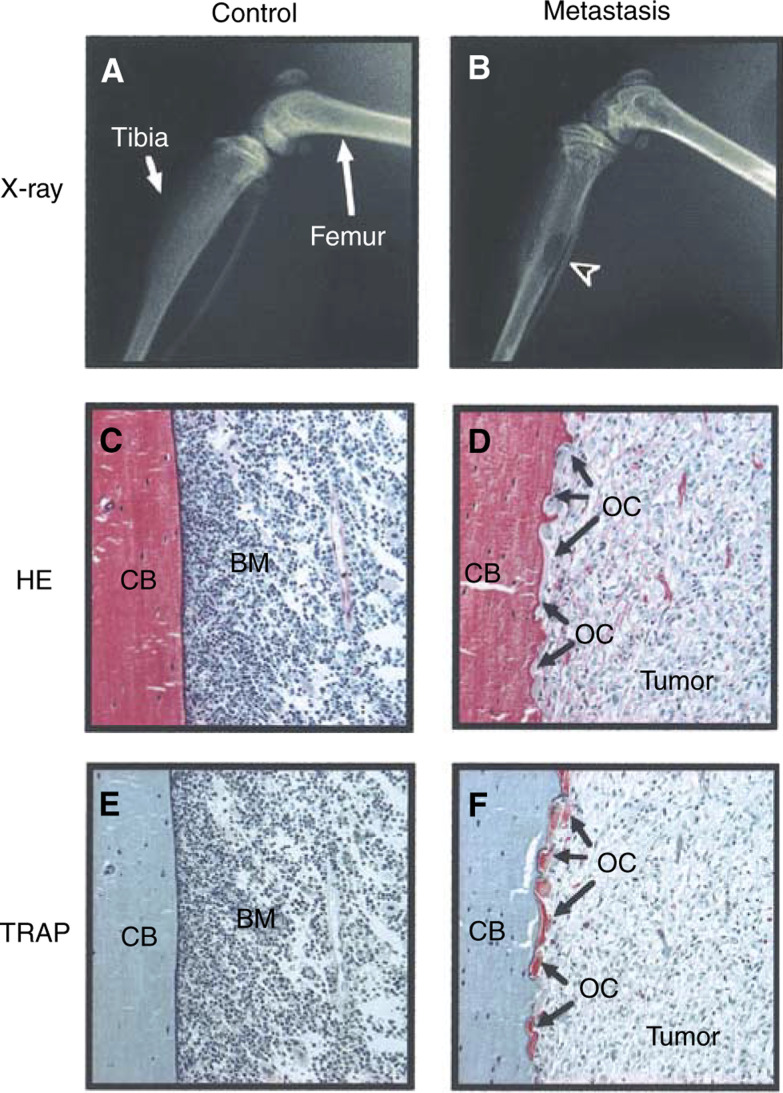
Representative radiographs and histological sections of osteolytic lesions in the hindlimbs of nude mice injected with MDA-231 cells. (**A**, **B**) Soft X-ray analyses of the hindlimbs of control mouse injected with PBS (A) and mouse injected with MDA-231 cells (B). Arrowhead indicates the osteolytic lesion because of metastasis in the tibia. (**C**, **D**) HE staining of histological sections of tibia collected from the mice shown in panels A and B, respectively. In the control section (C), bone marrow cells (BM) were normal and the cortical bone (CB) surface was smooth. In the metastasised section (D), tumour filled the bone marrow cavity and replaced the cellular elements. Arrows indicate osteoclasts (OC) on the cortical surface. (**E**, **F**) TRAP staining of the serial sections of panels C and D, respectively. In the metastasized section (F), TRAP-positive osteoclasts were aligned on the cortical surface.

We first performed histological analyses in bone with metastasis of breast cancer cells, MDA-231 cells, using a typical *in vivo* model. Radiographic examination demonstrated the development of metastatic osteolytic lesions in the femur and tibiae 6 weeks after the injection of MDA-231 cells in nude mice (Figure 1

). Tibiae were collected from these mice, and used for histological examination of the sections stained for HE and TRAP, a specific enzyme of osteoclasts. Control tibia showed normal bone marrow cells and a smooth cortical bone surface. The sections of tibia with osteolysis indicated that metastatic tumour cells filled the bone marrow cavity and that a number of TRAP-positive osteoclasts aligned on the surface of the cortical bone and resorbed bone to form resorption pits (Figure 1). The TRAP-staining of MDA-231 cells was negative in the section of the metastatic region (Figure 1).

### Expression of RANKL and MMPs in bone with metastasis

**Figure 2:**
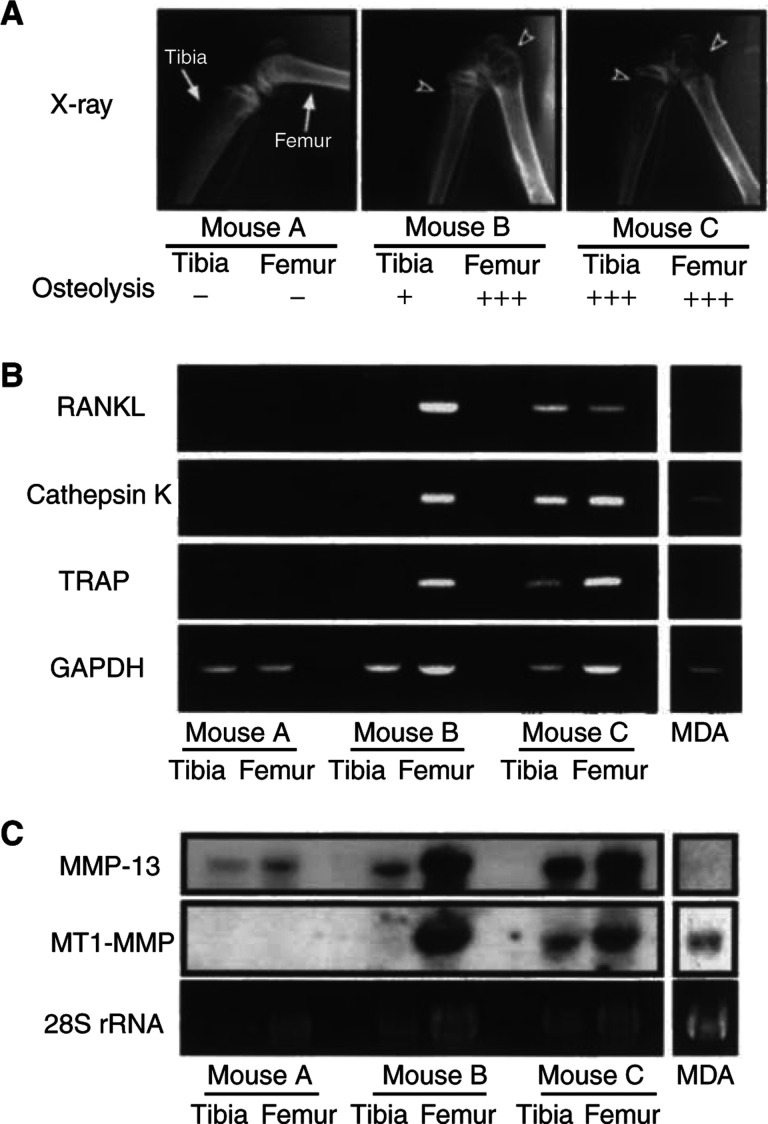
Expression of RANKL, cathepsin K, TRAP, MMP-13 and MT1-MMP mRNA in bone with metastasis of MDA-231 breast cancer. (**A**) Nude mice were injected with or without MDA-231 cells and the hindlimbs were subjected to soft X-ray analyses. Arrowheads indicate the osteolytic lesions because of metastasis in the femur and tibia. Mouse A is control mouse without injection of MDA-231 cells. Mouse B possesses slight metastasis in the tibia and severe metastasis in the femur. Mouse C possesses severe metastasis in both the femur and tibia. (**B**) Total RNA was collected from the femur and tibia shown in panel A, and mRNA expression of RANKL, cathepsin K and TRAP was analysed by RT–PCR. (**C**) Expression of MMP-13 and MT1-MMP mRNAs was analysed by Northern blotting using total RNA used in panel B. Note that marked expression of RANKL, cathepsin K, TRAP, MMP-13 and MT1-MMP mRNAs was detected only in bone with severe osteolysis because of bone metastasis.

Using the model of bone metastasis of MDA-231 cells, we examined the expression of RANKL and MMPs in bone with metastasis. Figure 2A

shows the radiographic data of mice used in this experiment. Mouse A is control without injection of MDA-231 cells. Mouse B has severe osteolysis in the distal femur and slight osteolysis in the proximal tibia. Mouse C shows severe osteolysis in both the femur and tibia. From these mice, we collected the tibia and femur to extract total RNA and used for Northern blot and RT–PCR analyses to detect the mRNA expression of RANKL and MMPs. It is well known that cathepsin K and TRAP are specifically expressed in osteoclasts (Minkin, 1982; Saftig *et al*, 1998). The expression of cathepsin K and TRAP was markedly elevated in the femur of mouse B and in the femur and tibia of mouse C, indicating that osteoclastic bone resorption was in progress in these bones (Figure 2B). The expression of RANKL mRNA was hardly detected in bone without metastasis, but bone with severe metastasis exhibited a marked expression of RANKL mRNA. MMP-13 is a critical enzyme for the degradation of type I collagen, and is known to be produced by osteoblasts treated with bone-resorbing factors (Kusano *et al*, 1998). The expression of MMP-13 mRNA was slightly detected in control bone in mouse A, and greatly elevated in bone with severe osteolysis in mice B and C (Figure 2C). MT1-MMP (MMP-14) is known to activate pro-MMP-2 and pro-MMP-13, and is essential for bone remodelling (Sato *et al*, 1994; Knauper *et al*, 1996; Holmbeck *et al*, 1999; Zhou *et al*, 2000). The expression of MT1-MMP mRNA was hardly detected in bone without metastasis, but bone with severe osteolysis showed a marked expression of MT1-MMP (Figure 2C). In RT–PCR using human-specific primers for RANKL and MMP-13, we detected no expression of RANKL and MMP-13 mRNAs not only in MDA cells cultured *in vitro* but also in bone with severe metastasis (data not shown). This suggests that the expression of RANKL and MMPs detected in bone with severe osteolysis was mostly derived from bone in nude mice.

### Coculture of MDA-231 cells in collagen gel and mouse calvaria

**Figure 3:**
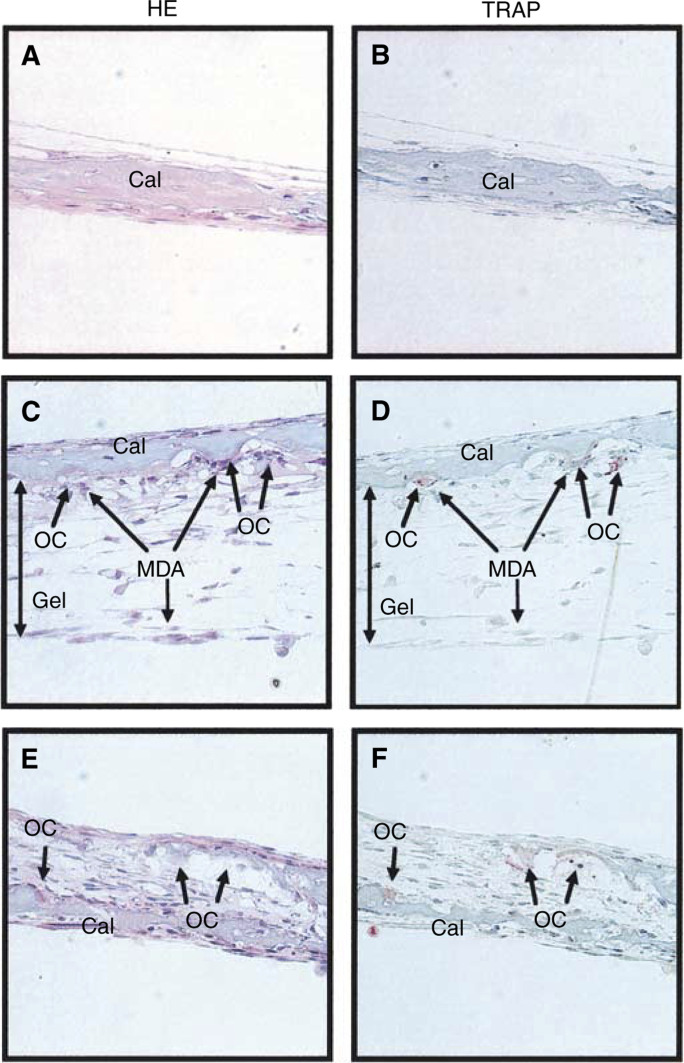
Histological view of coculture of mouse calvaria and MDA-231 breast cancer cells embedded in collagen gel. Calvariae were collected from 5-day-old mice, and cocultured for 5 days with MDA-231 cells, embedded in type I collagen gel to retain the position in culture plates. After the culture, the calvariae were fixed, sectioned and stained for HE and TRAP. (**A**, **B**) Section of the control calvaria. (**C**, **D**) Section of the coculture of calvaria and MDA-231 cells embedded in the gel. Osteoclasts and resorption pits were observed on the surface of the calvaria in contact with the gel containing MDA-231 cells. (**E**, **F**) As the positive control, calvariae were cultured for 5 days in the presence of 2 ng ml^−1^ of IL-1. Osteocalsts were observed inside the calvaria. Cal, calvaria; OC, osteoclasts; Gel, type I collagen gel; MDA, MDA-MD-231.

To examine the mechanism of tumour-induced bone resorption, we developed a new *in vitro* system using MDA-231 cells and calvaria collected from 5-day-old mice. MDA-231 cells were embedded in type I collagen gel to retain the position and cocultured for 5 days with mouse calvaria. Histological analysis showed that some MDA-231 cells in the collagen gel were in contact with the surface of the calvaria and that TRAP-positive osteoclasts formed resorption pits in the side of the calvaria attached to MDA-231 cells (Figure 3C, D

). In calvaria treated with 2 ng ml^−1^ of IL-1, on the other hand, osteoclasts resorbed calcified bone on the inside of the calvaria (Figure 3E, F).

### Bone-resorbing activity and expression of RANKL and MMPs in coculture of MDA cells and mouse calvaria

**Figure 4:**
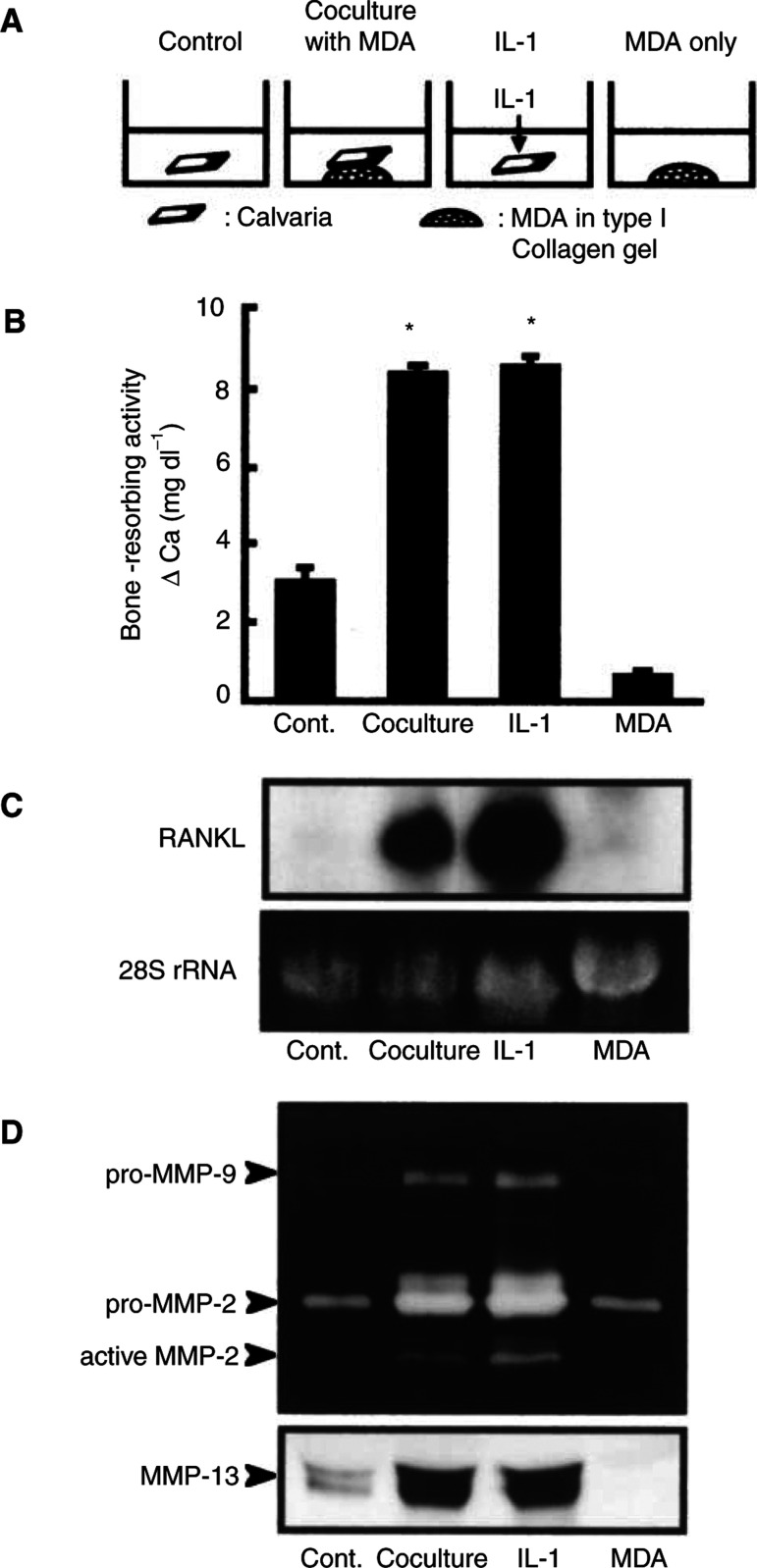
Bone-resorbing activity and expression of RANKL and MMPs in coculture of mouse calvaria and MDA-231 cells. (**A**) Illustrations of the experimental procedure of coculture of mouse calvaria and MDA-231 cells. Mouse calvariae were cocultured for 5 days with MDA-231 cells. As a positive control of bone resorption, calvariae were cultured for 5 days with 2 ng ml^−1^ of IL-1. MDA-231 cells (1 × 10^5^ cells) were cultured for 5 days as MDA-231 control without calvaria. (**B**) Conditioned media were collected, and the concentration of calcium in the medium was measured to monitor the bone-resorbing activity. Data are expressed as means±s.e.m. of six cultures. Significantly different from the control (^*^P<0.001). (**C**) Total RNA was extracted from calvaria-excluded MDA-231 cells, and Northern blotting was performed using ^32^P-labelled cDNA probes for RANKL as described in Materials and Methods. (**D**) Conditioned media were collected and gelatin zymography (upper panel) and Western blotting (lower panel) were performed as described in Materials and Methods. In the upper panel, gelatinase activities corresponding to pro-MMP-2, active-MMP-2 and pro-MMP-9 are indicated by arrows. In the lower panel, MMP-13 was detected using anti-mouse MMP-13 antibody. Note that the marked expression of RANKL, MMP-2 and MMP-13 was detected in the calvaria cocultured with MDA-231 cells, and that the levels were similar to those indued by IL-1.

Using the coculture system of MDA-231 cells and mouse calvaria, we examined the bone-resorbing activity by measuring medium calcium. When calvarial tissues were cocultured for 5 days with MDA-231 cells, the bone-resorbing activity was markedly elevated and the level was similar to that induced by 2 ng ml^−1^ of IL-1 (Figure 4B

). After the coculture, MDA-231 cells were removed from the calvarial tissues, and the expression of RANKL in the calvaria was examined by Northern blot analysis. In consistence with the data of bone-resorbing activity, the expression of RANKL mRNA was elevated in the calvaria cocultured with MDA-231 cells (Figure 4C). In this study, we also examined the expression of MMP-2, MMP-9 and MMP-13 by gelatin zymography and Western blot analysis. Gelatin zymography using the conditioned medium of the coculture revealed an enhanced production of pro-MMP-2, active-MMP-2 and pro-MMP-9. The production of MMP-13 was also elevated in the coculture with MDA-231 cells (Figure 4D). The level of MMP-2 and MMP-13 in the coculture was similar to that in the calvaria treated with 2 ng ml^−1^ of IL-1. In RT–PCR using human-specific primers for RANKL and MMP-13, the expression of their mRNAs could not be detected in both control MDA-231 cells and MDA-231 cells cocultured with calvaria (data not shown).

### Mechanisms of tumour-induced bone resorption

**Figure 5:**
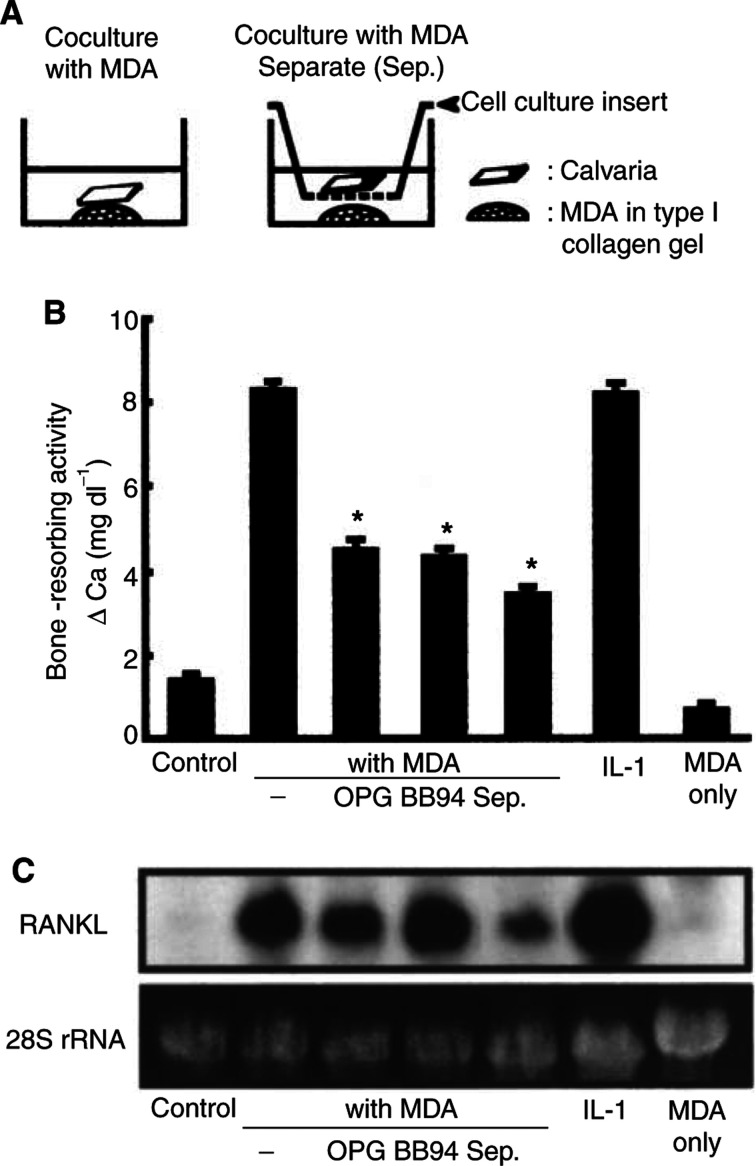
Roles of RANKL, MMPs and interaction between bone and tumour cells in bone-resorbing activity in coculture of calvariae and MDA-231 cells. (**A**) Illustrations of the experimental procedure of coculture and separate coculture using 5-day-old mouse calvaria and MDA-231 cells embedded in collagen gel. In separate cocultures, calvariae were separated from MDA-231 cells using cell culture inserts, which permeate soluble factors present in the culture media. (**B**) Bone-resorbing activity was measured by the increase in medium calcium using conditioned media collected on day 5 from coculture and separate coculture shown in panel A. In the cocultures, a subgroup was added to osteoprotegerin (OPG: 100 ng ml^−1^) or BB94 (10 *μ*M). Some calvariae were cultured with 2 ng ml^−1^ of IL-1 as positive control of bone resorption. MDA-231 cells (1 × 10^5^ cells) were cultured on plate for 5 days as control without calvaria (MDA only). Data are expressed as means±s.e.m. of six cultures. Significantly different from the value of coculture with MDA-231 (^*^P<0.001). (**C**) Total RNA was extracted from the calvaria used in the experiment shown in panel B, and Northern blotting was performed using ^32^P-labelled cDNA probe for RANKL. In the cocultures, MDA-231 cells in the collagen gel were removed from the calvaria prior to the extraction of total RNA from the calvaria.

To examine the role of RANKL and MMPs in bone resorption induced by MDA-231 cells, we added 100 ng ml^−1^ of OPG, a decoy receptor for RANKL, and 10 *μ*M BB94, a general inhibitor of MMPs, to the coculture of mouse calvaria and MDA-231 cells. The bone-resorbing activity induced by MDA-231 cells was significantly suppressed by adding OPG and BB94 on day 5 (Figure 5B

). Therefore, both RANKL and MMPs are involved in the mechanism of bone resorption induced by MDA-231 cells. Adding OPG and BB94 had little influence on the expression of RANKL mRNA in the coculture (Figure 5C). Adding OPG and BB94 showed no effect on growth of MDA-231 cells (data not shown).

Previous studies have shown the roles of soluble factors, such as PTHrP, produced by tumour cells in bone metastasis (Powell *et al*, 1991; Pederson *et al*, 1999). On the other hand, cell-to-cell interaction between tumour cells and stromal cells is thought to be essential for the production of cytokines and MMPs. To determine this point, the calvaria was separated from MDA-231 cells on day 5 in the coculture, as shown in Figure 5A. The bone-resorbing activity induced by MDA-231 cells was significantly suppressed when the calvaria was separated from MDA-231 cells (Figure 5B). The level of expression of RANKL mRNA was reduced in a separate coculture, but the level was still higher than that in controls (Figure 5C). These results suggest that cell-to-cell and cell-to-matrix interaction is essential for tumour-induced bone resorption, and that a soluble factor(s) produced by tumours is partially involved in the mechanism of bone resorption induced by breast cancer cells.

### Osteoclast formation induced by breast cancer cells *in vitro*

**Figure 6:**
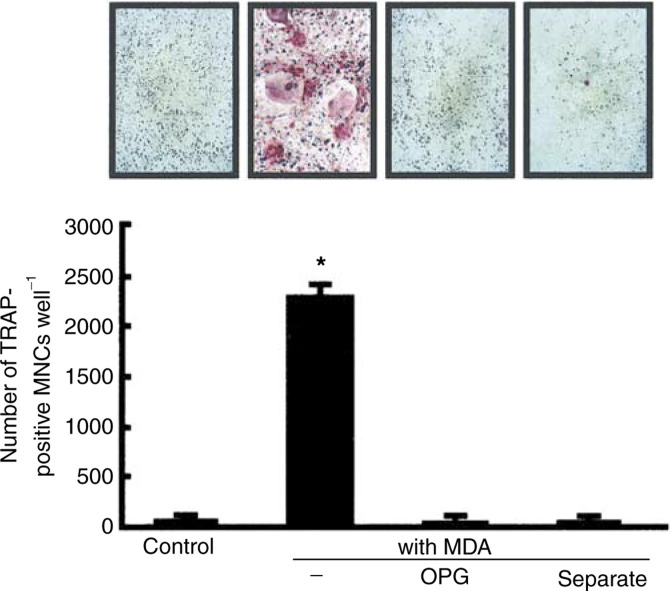
Osteoclast formation induced by MDA-231 cells in cocultures of mouse bone marrow cells and osteoblasts. Mouse bone marrow cells (2 × 10^6^) and osteoblasts (1 × 10^4^) were cocultured for 8 days with MDA-231 cells (1 × 10^4^) in the presence or absence of 100 ng ml^−1^ of OPG. In a separate coculture, MDA-231 cells were separated from bone marrow cells and osteoblasts using a cell culture insert. (**A**) Representative field of TRAP staining. (**B**) The number of TRAP-positive multinucleated cells (MNCs) containing three or more nuclei was counted. Data are expressed as the means±s.e.m. of three wells. Significantly different from the control (^*^P<0.001).

In place of osteolysis because of bone metastasis, numerous osteoclasts appear on the surface of bone *in vivo*, as shown in Figure 1. To test whether osteoclasts are formed by the presence of tumour cells *in vitro*, we added MDA-231 cells to the coculture of mouse bone marrow cells and osteoblasts. Usually, the addition of bone-resorbing factors is essential for osteoclast formation in the coculture system. However, as shown in Figure 6

, adding MDA-231 cells markedly induced osteoclast formation on day 8 without exogenous bone-resorbing factor. The osteoclast formation induced by MDA-231 cells was completely abolished by adding 100 ng ml^−1^ of OPG (Figure 6). In addition, when MDA-231 cells were separated from the bone marrow cells and osteoblasts using a cell culture insert, osteoclasts were not formed on day 8 in the coculture. Therefore, cell-to-cell interaction is essential for MDA-231-induced osteoclast formation.

### Adhesion to fixed MDA-231 cells induces the expression of RANKL in osteoblasts and osteoclast formation

**Figure 7:**
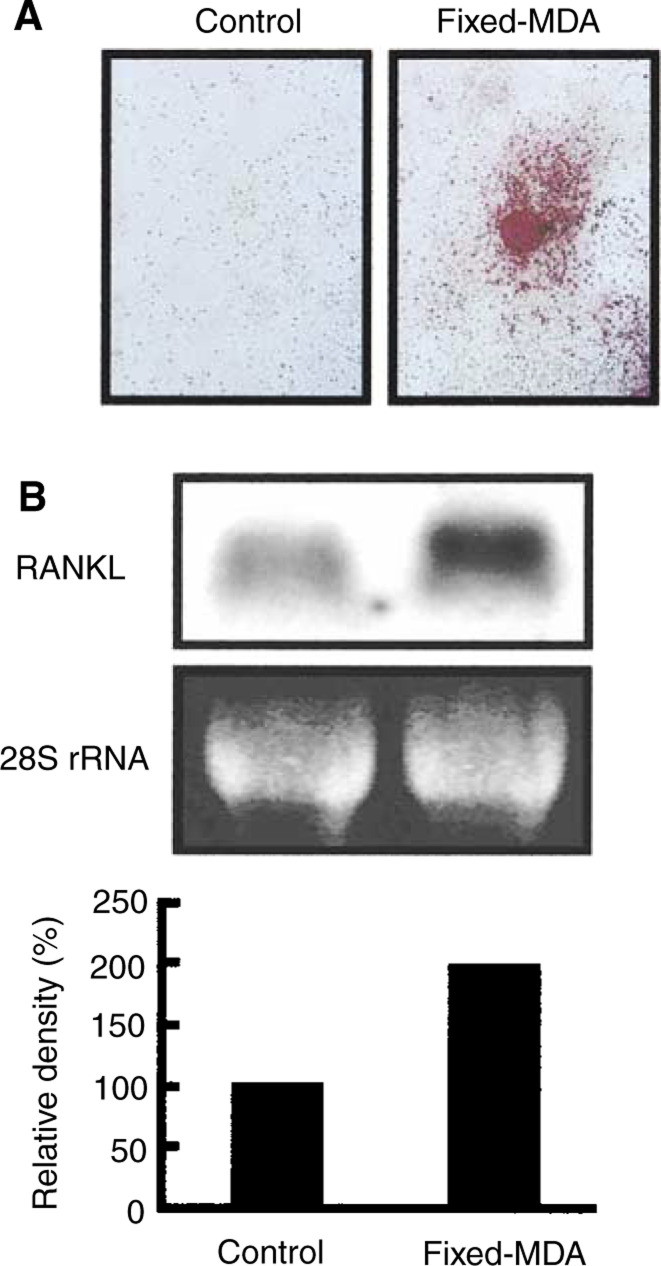
Cell-to-cell contact between osteoblasts and fixed-MDA-231 cells induces RANKL expression in osteoblasts, and osteoclast formation in coculture of bone marrow cells and osteoblasts. (**A**) Subconfluent MDA-231 cells were fixed with paraformaldehyde and washed with culture medium. Mouse bone marrow cells and osteoblasts were cocultured for 8 days on the layer of fixed-MDA-231 cells. Red colour staining shows representative field of TRAP staining. (**B**) Mouse osteoblasts were added to a culture dish (control) or the layer of fixed-MDA-231 cells (fixed-MDA), and cultured for 3 h to extract total RNA. Northern blotting was performed using ^32^P-labelled cDNA probe for RANKL. Signals in the upper panel were quantified using an image analyser as shown in the lower panel.

We next examined the effects of the adhesion of MDA-231 cells on the expression of RANKL mRNA in osteoblasts. MDA-231 cells were fixed with paraformaldehyde in the well, and mouse osteoblasts were added. At 3 h after adding the osteoblasts, the expression of RANKL mRNAs was elevated in the osteoblasts compared with the control osteoblasts without MDA-231 cells (Figure 7B

). These results indicate that cell-to-cell contact between MDA-231 cells and osteoblasts induces the expression of RANKL on the surface of osteoblasts. Indeed, adding fixed MDA-231 cells could induce osteoclast formation on day 8 in a coculture of bone marrow cells and osteoblasts without any exogenous bone-resorbing factor (Figure 7A).

## DISCUSSION

The present study demonstrated that the expression of RANKL and MMPs including MMP-13 and MT1-MMP was markedly elevated in bone with metastasis *in vivo*, and that the levels of expression of RANKL and MMPs were correlated with the extent of osteolysis (Figure 2). To examine the mechanism of bone resorption induced by tumour cells, we established a new coculture system of mouse calvaria and MDA-231 cells embedded in collagen gel, and found that MDA-231 cells markedly induced the bone-resorbing activity, the expression of RANKL in calvaria and the production of MMPs including MMP-2, MMP-9 and MMP-13 by calvaria (Figure 3 and Figure 4).

Recent studies have shown that RANKL expressed on the surface of osteoblasts is essential for osteoclast formation, and that osteoclast precursors, expressing RANK, recognise RANKL for their differentiation into mature osteoclasts (Anderson *et al*, 1997; Wong *et al*, 1997; Lacey *et al*, 1998; Yasuda *et al*, 1998). Adding MDA-231 cells to the coculture of mouse bone marrow cells and osteoblasts induced osteoclast formation without the requirement for any bone-resorbing factors (Figure 6). Using species-specific primers for RT–PCR, we distinguished the mRNA expression of human RANKL from that of mouse RANKL. MDA-231 cells showed no expression of human RANKL, whether bone cells were present or not (data not shown). Therefore, RANKL is expressed in host bone cells rather than MDA-231 cells. Zhang *et al* (2001) have reported that prostate cancer cells produce a soluble form of RANKL and directly induce osteoclastogenesis from osteoclast precursors. Although the tumour-induced osteoclastogenesis is mostly RANKL-dependent, the mechanism may be reliant on the tumour cells in each case.

OPG, an inhibitory molecule for osteoclastogenesis, binds to RANKL as a decoy receptor and suppresses the interaction between RANKL and RANK (Simonet *et al*, 1997; Tsuda *et al*, 1997; Kwon *et al*, 1998). Recent studies suggest that OPG may be a new therapeutic agent for bone loss associated with metabolic diseases such as osteoporosis and rheumatoid arthritis (Bucay *et al*, 1998; Kong *et al*, 1999; Kostenuik *et al*, 2001; Redlich *et al*, 2002). In the present study, OPG suppressed both bone resorption induced by MDA-231 cells in calvarial cultures and osteoclast formation induced by MDA-231 cells in bone marrow cultures. This is consistent with recent papers describing that tumour cells such as B16 melanoma and neuroblastoma induce osteoclast formation by a RANKL-dependent mechanism *in vitro* (Chikatsu *et al*, 2000; Michigami *et al*, 2001).

Bone resorption is mediated by not only osteoclast differentiation, but also matrix degradation. Cathepsin K and MMP-9 are selectively produced by osteoclasts in bone, but MMP-2, MMP-13 and MT1-MMP are mainly expressed in osteoblasts (Meikle *et al*, 1992; Reponen *et al*, 1994; Kinoh *et al*, 1996; Kusano *et al*, 1998). We have reported that bone-resorbing factors such as IL-1 and PGE2 markedly induced the expression of MMPs including MMP-2, MMP-3 and MMP-13, and that the induction of MMPs was associated with an increase in bone-resorbing activity in mouse calvarial cultures (Kusano *et al*, 1998; Miyaura *et al*, 2000). Other reports have also shown that MMPs are involved in the process of bone resorption (Hill *et al*, 1994). In the present study, we detected a marked expression of MMP-13 and MT1-MMP in bone with metastasis *in vivo* (Figure 2). In coculture of mouse calvaria and MDA-231 cells, the production of MMP-2, MMP-9 and MMP-13 was markedly enhanced, and adding BB94, a general inhibitor of MMPs, significantly suppressed the bone-resorbing activity elicited by MDA-231 cells (Figure 4 and Figure 5). Therefore, MMPs produced by osteoblasts and osteoclasts may contribute to the matrix degradation at the site of tumour-induced osteolysis.

MMPs are known to be involved in several processes of cancer metastasis. Infiltration of tumour cells from the blood vessels and invasion into metastatic organs are regulated by MMPs such as MMP-2 and MT1-MMP produced by host cells as well as tumour cells (MacDougall and Matrisian, 1995; Nakahara *et al*, 1997; Westermarck and Kahari, 1999). Yoneda *et al* (1997) have reported that osteolytic bone metastasis of breast cancer could be inhibited by combined treatment with bisphosphonate, a specific inhibitor of osteoclastic bone resorption, and tissue inhibitor of MMP (TIMP)-2, a natural inhibitor of MMPs. Since previous studies have shown that recombinant TIMP-1 and TIMP-2 inhibited bone resorption *in vitro* (Hill *et al*, 1993), TIMP-2 may suppress bone metastasis by inhibiting both tumour invasion and bone resorption.

Previous reports have shown that cancer cells produce various humoral factors such as PTHrP and bone-resorbing cytokines (Powell *et al*, 1991; Pederson *et al*, 1999). It is possible that a soluble factor(s) produced by tumour cells is involved in the mechanism of tumour-induced osteolysis. PTHrP was reported to contribute to breast cancer-mediated osteolysis (Powell *et al*, 1991), and anti-PTHrP neutralising antibody was shown to be effective in osteolysis (Guise *et al*, 1996). Mancino *et al* (2001) have reported that MDA-231 cells stimulate osteoclast formation by secreting macropahge colony-stimulating factor (M-CSF) and by enhancing the expression of RANKL in stromal support cells. It is well known that M-CSF produced by osteoblastic stromal cells is essential for osteoclast formation. In the present study, the bone-resorbing activity induced by MDA-231 cells was significantly suppressed when the calvaria was separated from MDA-231 cells, but the activity was still higher than that of controls in calvarial cultures (Figure 5). Therefore, bone resorption induced by MDA-231 cells may be due in part to humoral factors such as PTHrP and M-CSF produced by MDA-231 cells. On the other hand, cell-to-cell interaction seems to play an important role in osteoclast formation induced by MDA-231 cells, because osteoclast formation was completely suppressed when MDA-231 cells were separated from bone marrow cells (Figure 6).

To examine the role of cell-to-cell interaction between tumour cells and host cells in osteoclast formation, we fixed MDA-231 cells before the contact with bone marrow cells. Fixed MDA-231 cells induced osteoclast formation in bone marrow cultures and the expression of RANKL in mouse osteoblasts (Figure 7), indicating that molecule(s) expressed on the surface of MDA-231 cells may be recognised by osteoblasts. Osteoblasts as well as tumour cells express various integrins, and integrin signals are known to regulate osteoclast formation (Matsuura *et al*, 1996; Akatsu *et al*, 1998; Pommerenke *et al*, 2002). The molecules involved in cell-to-cell recognition between MDA-231 cells and osteoblasts and the signal transduction for RANKL expression in osteoblasts are under investigation in our laboratory.

In conclusion, our present study demonstrated that the expression of RANKL and MMPs was markedly elevated in bone with osteolytic metastasis *in vivo*, and that breast cancer cells induced osteoclast formation by a mechanism involving cell-to-cell interaction between cancer cells and osteoblasts. Both RANKL-induced osteoclastogenesis and MMP-dependent matrix degradation may contribute to osteolysis because of bone metastasis.
